# Association between trait mindfulness and self-efficacy in sports-disadvantaged college students in China: the chain mediating role of exercise motivation and persistence

**DOI:** 10.3389/fpsyg.2025.1636692

**Published:** 2025-09-19

**Authors:** Kuo Xu, Lin Zhu, Yun Li, Yang Cao, Guodong Zhang

**Affiliations:** 1 Institute of Sport Science, College of Physical Education, Southwest University, Chongqing, China; 2 Biquan Primary School, Chongqing, China; 3 Clinical Epidemiology and Biostatistics, Faculty of Medicine and Health, School of Medical Sciences, Örebro, Sweden; 4 Unit of Integrative Epidemiology, Institute of Environmental Medicine, Karolinska Institutet, Stockholm, Sweden; 5 International College, Krirk University, Bangkok, Thailand

**Keywords:** exercise motivation, exercise persistence, trait mindfulness, self-efficacy, sports-disadvantaged college students, chain mediation effect

## Abstract

**Objective:**

This study investigates the effect of trait mindfulness on self-efficacy in sports-disadvantaged Chinese college students, focusing on the sequential mediating roles of exercise motivation and persistence, to inform interventions that enhance physical activity and psychological well-being.

**Method:**

A randomly selected sample of 600 sports-disadvantaged college students in China was surveyed, yielding 588 valid responses (male = 296, 50.3%; female = 292, 49.7%). Participants ranged in age from 18 to 23 years (*M* = 20.06, SD = 1.44). Among them, 45.1% reported smoking and 58.8% reported drinking. The assessment instruments included the Trait Mindfulness Scale, the Exercise Motivation Scale, the Self-Efficacy Scale, and the Exercise Persistence Scale. Data were analyzed using SPSS 26.0 and the PROCESS macro (version 4.1).

**Results:**

Significant correlations were identified among trait mindfulness, self-efficacy (*r* = 0.581, *p* < 0.01), exercise motivation (*r* = 0.585, *p* < 0.01), and exercise persistence (*r* = 0.545, *p* < 0.01) within the group of sports-disadvantaged college students. Exercise motivation was significant correlated with both exercise persistence (*r* = 0.592, *p* < 0.01) and self-efficacy (*r* = 0.679, *p* < 0.01). Exercise persistence also showed a significant correlation with self-efficacy (*r* = 0.639, *p* < 0.01). In the effect of trait mindfulness on self-efficacy among sports-disadvantaged college students, both exercise motivation (β = 0.224, 95% confidence interval (CI): [0.176, 0.277]) and exercise persistence (β = 0.100, 95% CI [0.067, 0.136]) demonstrated significant mediating effects. Furthermore, exercise motivation and exercise persistence exhibited a significant chain mediating effect in the influence of trait mindfulness on self-efficacy (β = 0.072, 95% CI [0.050, 0.099]).

**Conclusion:**

This study offers critical theoretical insights into the interplay between trait mindfulness, exercise behavior, and self-efficacy among sports-disadvantaged college students. It provides a foundation for developing targeted interventions and practical guidance for universities in enhancing sports education, optimizing resource allocation, and establishing psychological support systems. By addressing the unique needs of this population, institutions can foster greater physical engagement and psychological well-being, thereby advancing overall student health.

## 1 Introduction

In recent decades, China’s rapid socioeconomic transformation has profoundly reshaped its educational landscape, yielding unprecedented gains in access, quality, and national human capital development (Xiao et al., 2024; Zhao et al., 2024). However, this progress has also intensified academic pressure, lifestyle competition, and psychosocial stress among university students (Sharp and Theiler, 2018; Liu et al., 2024). Mounting empirical evidence reveals that approximately 21.48% of college students are at risk of depression, while 45.28% face significant anxiety symptoms (Li et al., 2022). making mental health an increasingly urgent concern in Chinese higher education (Wu et al., 2020).

The term sports-disadvantaged college students is widely used in higher education to designate a vulnerable subgroup of the student population who, due to chronic diseases, physical disabilities or dysfunctions, or recovery from illness or surgery, are unable to participate in regular or high-intensity physical exercise comparable to their peers (Liska et al., 2024; Wang et al., 2024b). These students typically demonstrate limited physical fitness, insufficient sports skills, and low levels of participation in physical activities (Bize et al., 2007), accounting for an estimated 19.1% of the college population (Stallman, 2010). Beyond these physical constraints, they frequently encounter social and psychological marginalization within physical activity contexts, manifesting in low self-esteem, diminished self-confidence, avoidance behaviors, and mental health difficulties such as anxiety and depression (Galanakis et al., 2016; Coudevylle et al., 2011). Moreover, the interaction between physical illness or disability and psychological strain often produces a reinforcing cycle: physical inactivity exacerbates feelings of inferiority and psychological vulnerability, which in turn further impedes their overall health, social integration, and well-being (Kocjan et al., 2024).

To interrupt this cycle, it is essential to clarify the mechanisms through which internal psychological traits are translated into adaptive health behaviors and improved mental health outcomes. The present study is grounded in three prominent behavior change frameworks: Social Cognitive Theory (SCT) (Young et al., 2014; Petosa et al., 2003), Self-Determination Theory (SDT) (Teixeira et al., 2012; Ng et al., 2012), and the Health Action Process Approach (HAPA) (Schwarzer, 2016; Joveini et al., 2020). These theoretical perspectives collectively conceptualize health behavior as the outcome of dynamic interactions among cognitive beliefs, motivational processes, and sustained behavioral engagement.

Central to SCT is self-efficacy, defined as an individual’s belief in their capability to perform behaviors necessary to produce desired outcomes (Anderson et al., 2007). It has consistently been linked to psychological well-being, academic persistence, and physical health (Rüppel et al., 2015). While traditionally conceptualized as a precursor to action, recent empirical studies emphasize a bidirectional or outcome-oriented role. Positive behavioral experiences, particularly successful engagement in physical activity, have been shown to reinforce and enhance self-efficacy (Alp Christ et al., 2024). For sports-disadvantaged students, whose baseline self-efficacy is often fragile, experiences of sustained exercise and overcoming bodily limitations may represent a primary source of psychological growth (Day and Wadey, 2017). Thus, in this context, self-efficacy is conceptualized as a dependent outcome shaped by psychological traits and behavioral mediators.

Recent literature highlights trait mindfulness as a dispositional capacity to remain attentively aware and nonjudgmentally present. It has been identified as a protective factor for both mental health and physical engagement (Rau and Williams, 2016; Rau and Williams, 2016). Through mechanisms of emotion regulation, attentional control, and decreased cognitive reactivity, trait mindfulness fosters psychological resilience and stress tolerance, particularly among vulnerable populations (Prakash et al., 2015; Paul et al., 2013). It also enhances individuals’ capacity to cope with discomfort during exercise, thereby promoting physical activity initiation and engagement. Accordingly, we propose Hypothesis 1 (H1): Trait mindfulness positively predicts self-efficacy among sports-disadvantaged college students.

Beyond this direct pathway, Self-Determination Theory (SDT) provides a robust framework for understanding how mindfulness fosters intrinsic motivation for exercise. Mindfulness has been shown to promote autonomous regulation by enhancing self-awareness, aligning behaviors with personal values, and reducing dependence on external rewards (Schuman-Olivier et al., 2020; Vago and David, 2012; Ryan et al., 2021). These effects are particularly relevant for sports-disadvantaged individuals, whose prior experiences may have eroded intrinsic interest and volitional control. Through the internalization of health goals and increased competence perception, mindfulness supports motivational restoration. Thus, we propose Hypothesis 2 (H2): Exercise motivation mediates the relationship between trait mindfulness and self-efficacy.

However, intention or motivation alone is often insufficient for behavior maintenance. The Health Action Process Approach (HAPA) underscores the need for volitional processes such as action planning, behavioral regulation, and adherence to bridge the gap between motivation and sustained physical activity (Schwarzer, 2016). Exercise adherence, defined as the ability to maintain regular physical activity over time despite obstacles, emerges as a critical mediating factor in this regard (Mcauley and Courneya, 1993). Empirical findings show that mindfulness facilitates adherence by fostering acceptance of discomfort, enhancing cognitive flexibility, and strengthening regulatory endurance (Lindsay et al., 2018). These factors are essential for sustaining long-term health behavior in the absence of external incentives. Moreover, the positive feedback generated by continued exercise further reinforces self-efficacy, particularly in populations that previously lacked such mastery experiences (Rodrigues et al., 2023; Wuepper and Lybbert, 2017). Therefore, we hypothesize Hypothesis 3 (H3): Exercise adherence mediates the relationship between exercise motivation and self-efficacy.

**Figure 1:**
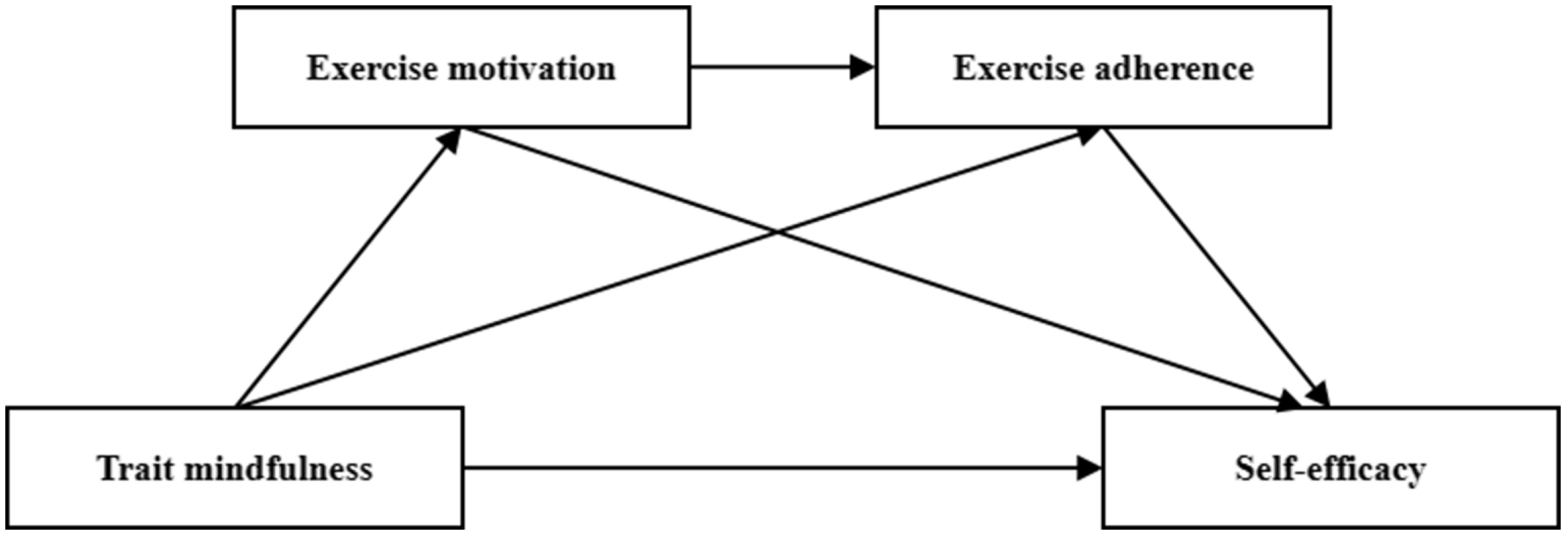
The mediating role model of exercise motivation and exercise persistence in the relationship between trait mindfulness and self-efficacy.

Taken together, this study proposes a chain mediation model in which trait mindfulness enhances exercise motivation, which in turn promotes greater exercise adherence, ultimately leading to increased self-efficacy. This sequential pathway reflects a theoretically coherent and empirically supported integration of cognition, motivation, and behavior. It also offers valuable insights into psychological intervention strategies for enhancing health equity among sports-disadvantaged college students. Therefore, we propose Hypothesis 4 (H4): Exercise motivation and adherence sequentially mediate the relationship between trait mindfulness and self-efficacy among sports-disadvantaged college students. The proposed conceptual framework is illustrated in Figure 1.

## 2 Research methodology

### 2.1 Research design and participants

This study employed a cross-sectional design, collecting data from sports-disadvantaged college students through a questionnaire survey. Data collection was conducted in April 2023 across six universities in Southwest China.

A stratified random sampling method was used to ensure representation across different universities and academic disciplines. Each university was treated as a stratum, within which students were randomly selected to participate. This approach aimed to enhance the representativeness of the sample and reduce sampling bias.

A total of 600 questionnaires were distributed, and 588 valid responses were obtained, resulting in an effective response rate of 98%. The sample size was determined based on the principle of ensuring statistical power for structural equation modeling analysis, referencing the commonly recommended sample-to-parameter ratio of at least 10:1. Given the number of parameters to be estimated in this study’s model, a minimum of 300 respondents was deemed sufficient; hence, the final sample size of 588 met and exceeded this requirement, ensuring robust and reliable statistical analysis.

The research instruments included the Trait Mindfulness Scale, Exercise Motivation Scale, Exercise Adherence Scale, and Self-Efficacy Scale. All questionnaires were completed online, with detailed instructions provided beforehand to ensure clarity and standardization of responses.

To ensure the quality of the data and the appropriateness of respondents, this study established scientifically grounded inclusion and exclusion criteria based on national standards and psychometric considerations.

- Inclusion criteria:

Participants were included if they met the definition of being “sports-disadvantaged,” operationalized as those scoring below 60 points on the *National Physical Fitness Standards for Chinese Students (College Level)*. This threshold is commonly used in education and health research to identify individuals with low physical fitness levels (Erwin, 2008). The criteria include multiple indicators such as cardiovascular endurance, muscular strength, flexibility, and body composition, which provide a multidimensional and standardized framework for evaluating physical disadvantage. Therefore, using this cutoff score offers a valid and objective method for identifying the target population in a manner consistent with national health benchmarks.

(b–e) Exclusion criteria:

To improve data reliability, the following responses were excluded:

- Questionnaires completed in less than 300 s, indicating inattentive or rushed participation
- Incomplete questionnaires
- Responses displaying patterned or uniform answering.
- Responses with logical inconsistencies across scales, suggesting careless or disengaged answering.

Regarding instrument validity, all scales employed in this study (Trait Mindfulness Scale, Exercise Motivation Scale, Exercise Adherence Scale, and General Self-Efficacy Scale) have been widely validated in Chinese university student populations (Luszczynska et al., 2005; Zhang et al., 2024; Tao et al., 2024). Each scale’s internal consistency was reassessed in the present sample, and all Cronbach’s α coefficients exceeded 0.80, indicating strong reliability. Moreover, confirmatory factor analysis (CFA) was conducted to verify structural validity, and the model fit indices met accepted thresholds, supporting the continued use of these instruments in the current research context.

In terms of analytical depth, while some scales were used as composite unidimensional variables for initial analyses, the multidimensional structures of key scales—particularly exercise motivation and trait mindfulness were preserved in mediation and moderation models. We acknowledge the potential theoretical richness offered by these subdimensions; therefore, in subsequent structural equation modeling (SEM) analyses, we explored them as separate mediators and independent variables. This approach revealed more nuanced psychological pathways and yielded greater explanatory power.

To ensure ethical compliance, the study strictly followed the principles of the 1964 Helsinki Declaration and its later amendments. All participants received informed consent documents that clearly outlined the study’s aims, procedures, and any potential risks. Participation was entirely voluntary, and respondents retained the right to withdraw at any time without penalty or loss of benefit.

### 2.2 Research instruments

#### 2.2.1 Trait mindfulness scale

This study utilized the Chinese Revised Version of the Mindful Attention Awareness Scale (MAAS), developed by Chen et al. (2012), to assess participants’ levels of trait mindfulness. This unidimensional scale comprises 15 items rated on a 6-point Likert scale (1 = Almost Always, 2 = Very Frequently, 3 = Somewhat Frequently, 4 = Somewhat Infrequently, 5 = Very Infrequently, 6 = Rarely). Higher scores indicate greater levels of trait mindfulness among participants. This scale has been tested for reliability and validity, demonstrating its suitability for use with Chinese college students. However, given its limited application among sports-disadvantaged college students in China, this study conducted an additional reliability test on the scale. In this context, the Cronbach’s α coefficient for the scale was 0.880, indicating high consistency.

#### 2.2.2 Self-efficacy scale

This study adopted the Chinese version of the General Self-Efficacy Scale (GSES), originally developed by Schwarzer et al. and subsequently translated and revised by Wang et al. The scale is a unidimensional instrument comprising 10 items, specifically designed to assess individuals’ perceptions of their general self-efficacy.

A 4-point Likert scale was employed in this study (1 = Completely Incorrect, 2 = Partially Correct, 3 = Mostly Correct, 4 = Completely Correct), with higher scores indicating stronger perceived self-efficacy. Although a 5-point scale is commonly used in similar research, the 4-point format was intentionally selected for two primary reasons. First, it eliminates the neutral midpoint, compelling respondents to express a clearer stance, which helps reduce central tendency bias and improves the discriminatory power of the scale. Second, empirical studies have demonstrated the psychometric soundness of the 4-point version within comparable populations. For instance, Wang et al. (2024a) validated the 4-point Chinese version of the GSES among university students in China and reported satisfactory reliability and construct validity, further supporting the appropriateness of its use in the current study (Zeng et al., 2022).

The Chinese version of the GSES has been widely applied in higher education research contexts, consistently showing robust reliability and validity. In the present study, the scale demonstrated good internal consistency, with a Cronbach’s α coefficient of 0.827, indicating that it is a reliable tool for measuring self-efficacy among Chinese college students.

#### 2.2.3 Exercise motivation scale

This study used the Revised Motivation for Physical Activity Scale (MAM-R), adapted by Chen et al. (2013), to assess participants’ levels of exercise motivation. The scale’s applicability among Chinese college students has been verified, demonstrating that it effectively measures exercise motivation in this population. The scale consists of 15 items divided into five dimensions: Health Motivation (items 1–3), Appearance Motivation (items 4–6), Enjoyment Motivation (items 7–9), Competence Motivation (items 10–12), and Social Motivation (items 13–15). The scoring uses a 5-point Likert scale (1 = Strongly Disagree, 2 = Disagree More, 3 = Neutral, 4 = Agree More, 5 = Strongly Agree). Higher scores indicate a greater level of exercise motivation in the participants. In this study, the Cronbach’s α coefficient for the overall scale was 0.885, with Cronbach’s α coefficients for each subdimension ranging between 0.811 and 0.845.

#### 2.2.4 Exercise adherence scale

This study used the Exercise Adherence Scale (EAS), developed by Wang et al. (2016), to assess participants’ levels of exercise adherence. The scale comprises 14 items divided into three dimensions: Exercise Behavior (items 1–4), Effort Investment (items 5–9), and Emotional Experience (items 10–14). A 5-point Likert scale was used for scoring (1 = Completely Inconsistent, 2 = Mostly Inconsistent, 3 = Somewhat Consistent, 4 = Relatively Consistent, 5 = Completely Consistent). Higher scores indicate a greater level of personal exercise adherence. This scale has undergone reliability and validity testing among Chinese college students, demonstrating strong applicability. In this study, the Cronbach’s α coefficient for the overall scale was 0.848.

### 2.3 Statistical analysis

Data entry for the collected questionnaires was performed using Excel 2013, followed by relevant data analysis. First, descriptive statistics were conducted on the collected data. After confirming a normal distribution, Pearson correlation analysis was used to examine the relationships between variables. When all four variables showed significant pairwise correlations, a mediation effect analysis was conducted. The mediation effect analysis was conducted using the PROCESS macro (version 4.1) for SPSS, developed by Hayes (2013). To explore the relationship between independent variable (trait mindfulness) and dependent variable (self-efficacy), which may be affected by mediator variable (exercise motivation) and mediator variable (exercise persistence). In PROCESS, Model 6 was used for mediation analysis, with 5,000 bootstrap resamples, utilizing bias-corrected percentile bootstrap confidence intervals (CIs) to assess effect sizes. A mediation effect was considered significant if the 95% CI did not include zero. In the mediation effect analysis, participants’ age, gender, smoking status, and drinking habits were included as covariates to control for potential confounding.

Additionally, to examine the potential presence of common method bias, Harman’s single-factor test was conducted using exploratory factor analysis on all measured variables. The results indicated that nine factors had eigenvalues greater than 1, and the first unrotated factor accounted for only 30.91% of the total variance—below the critical threshold of 40%—suggesting that common method bias was not a serious concern in this study.

## 3 Results

### 3.1 Common method bias test

In this study, Harman’s single-factor test was applied to all variables using exploratory factor analysis. The results revealed nine factors with eigenvalues exceeding 1. Notably, the most significant factor accounted for only 30.91% of the variance, below the 40% threshold, indicating the absence of common method bias in this research.

### 3.2 Descriptive statistics and correlation analysis

This study included 588 participants for analysis. Male participants comprised 50.3% of the sample. The participants ranged in age from 18 to 23. Additionally, a relatively high proportion of participants reported smoking, accounting for 30–45% of the total sample, with a balanced distribution between those who consume alcohol and those who abstain. Detailed results are presented in Table 1.

**Table 1:** Characteristics of the participants.

Categorical variables	Category	*N*	Percentage (%)
Sex	Male	296	50.3
	Female	292	49.7
Age	18	93	15.8
	19	137	23.3
	20	147	25.0
	21	95	16.2
	22	85	14.5
	23	31	5.3
Smoking	Yes	265	45.1
	No	323	54.9
Drinking	Yes	346	58.8
	No	242	41.2

The correlation analysis results (Table 2) indicate that trait mindfulness is significantly positively correlated with exercise motivation (*r* = 0.585, *p* < 0.01), exercise adherence (*r* = 0.545, *p* < 0.01), and self-efficacy (*r* = 0.581, *p* < 0.01). Additionally, exercise motivation is positively correlated with exercise adherence (r = 0.592, *p* < 0.01) and self-efficacy (*r* = 0.679, *p* < 0.01). Finally, exercise adherence shows a significant positive correlation with self-efficacy (*r* = 0.639, *p* < 0.01). All four outcome variables are positively correlated, which serves as an essential prerequisite for the subsequent analysis of the chain mediation effect.

**Table 2:** Descriptive statistics and correlation analysis.

Variables	Mean	SD	Pearson’s correlation coefficient			
			Trait mindfulness	Exercise motivation	Exercise adherence	Self-efficacy
Trait mindfulness	3.40	1.01	1			
Exercise motivation	3.33	0.91	0.585**	1		
Exercise adherence	3.13	0.81	0.545**	0.592**	1	
Self-efficacy	2.75	0.66	0.581**	0.679**	0.639**	1

SD, Standard deviation; **p < 0.01.

### 3.3 Mediation effect test of exercise motivation and exercise adherence

The regression analysis results (Table 3) reveal that trait mindfulness positively predicts self-efficacy among sports-disadvantaged college students (β = 0.591, *p* < 0.001), confirming hypothesis H1. When mediating variables are introduced, the direct effect of trait mindfulness on participants’ self-efficacy diminishes (β = 0.195, *p* < 0.001), while trait mindfulness significantly predicts both exercise motivation (β = 0.583, *p* < 0.001) and exercise adherence (β = 0.330, *p* < 0.001). Additionally, exercise motivation significantly predicts exercise adherence (β = 0.406, *p* < 0.001) and self-efficacy (β = 0.385, *p* < 0.001), and exercise adherence significantly predicts self-efficacy (β = 0.303, *p* < 0.001). These findings suggest that exercise motivation and adherence act as mediators, forming a chain of mediation in the influence of trait mindfulness on self-efficacy in this group.

**Table 3:** Regression analysis of chain mediation effects for exercise motivation and exercise persistence (*N* = 588).

Regress equation		Fitting index			Significance		
Outcome variable	Predictor variable	*R*	*R* ^2^	*F*	β	SE	*t*
Self-efficacy	Trait mindfulness	0.594	0.353	63.496^***^	0.591	0.034	17.450^***^
	Age				0.078	0.023	3.358^***^
	Sex				0.005	0.068	0.070
	Smoking tipple				−0.176 −0.173	0.102 0.068	−1.722 −0.253
Exercise motivation	Trait mindfulness	0.596	0.354	64.007^***^	0.583	0.034	17.226^***^
	Age				0.062	0.023	2.681^*^
	Sex				−0.123	0.068	−1.868^**^
	Smoking tipple				−0.111 −0.019	0.102 0.068	−1.087 −0.271^*^
Exercise adherence	Trait mindfulness	0.667	0.445	77.566^***^	0.330	0.039	8.542^***^
	Exercise motivation				0.406	0.039	10.557^***^
	Age				0.086	0.023	3.950^**^
	Sex				0.205	0.063	3.248
	Smoking tipple				−0.050 0.162	0.095 0.063	−0.519 2.557^*^
Self-efficacy	Trait mindfulness	0.755	0.570	109.968^***^	0.195	0.036	5.419^***^
	Exercise motivation				0.385	0.037	10.401^***^
	Exercise adherence				0.303	0.037	8.292^***^
	Age				0.021	0.019	1.060
	Sex				0.007	0.056	0.125
	Smoking tipple				−0.105 −0.057	0.084 0.056	−1.255 −1.016

SE, standard error, β = point estimate of effect size, *p < 0.05, **p < 0.01, ***p < 0.001.

**Figure 2:**
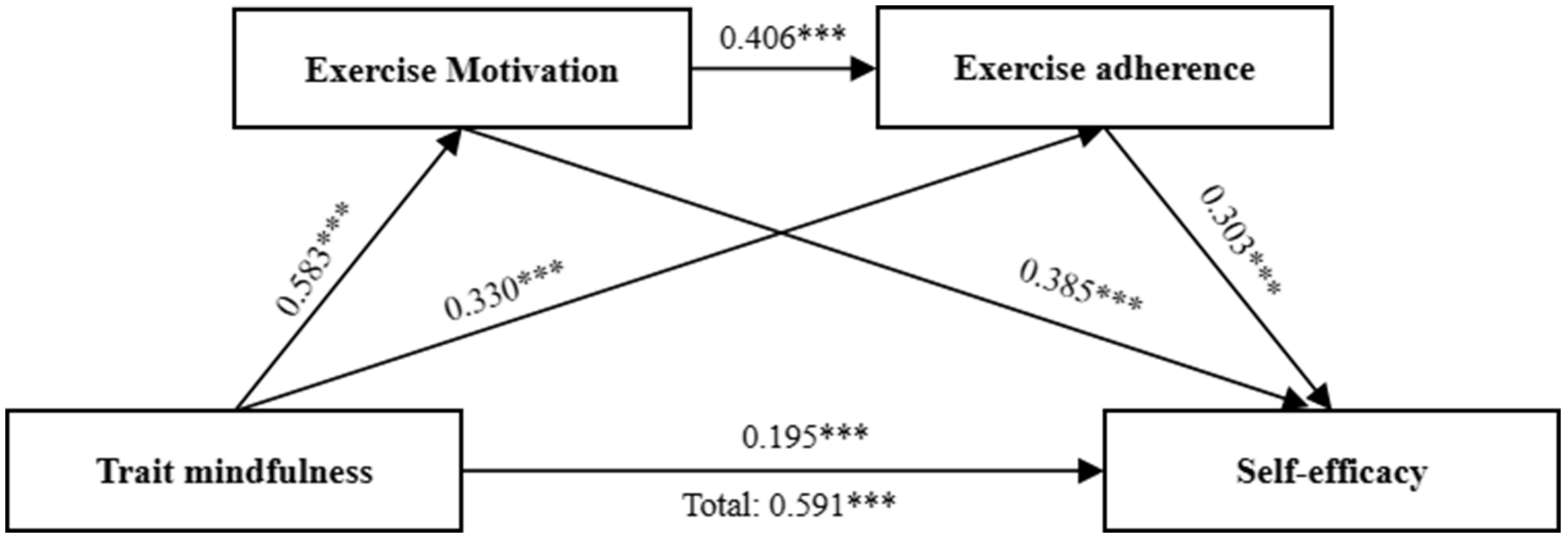
Chain mediation effects of exercise motivation and persistence on trait mindfulness and self-efficacy. ***p < 0.001.

The mediation analysis results (Table 4; Figure 2) show a significant direct effect of trait mindfulness on self-efficacy within the mediation model (β = 0.195, 95% CI [0.125, 0.266]). Additionally, significant indirect effects were observed, with all three mediation pathways reaching significance:

**Table 4:** The mediating role of exercise motivation and persistence (*N* = 588).

Model	Effect size	SE	95% Bootstrapping CI	*p*-value
Direct effect	0.195	0.036	0.125–0.266	<0.001
Total indirect	0.396	0.030	0.340–0.455	<0.001
Indirect effect 1	0.224	0.026	0.176–0.277	<0.001
Indirect effect 2	0.100	0.018	0.067–0.136	<0.001
Indirect effect 3	0.072	0.013	0.050–0.099	0.002
Total effect	0.591	0.034	0.525–0.658	< 0.001

SE, Standard error; Indirect effect 1 = Trait mindfulness → Exercise motivation → self-efficacy; Indirect effect 2 = Trait mindfulness → Exercise persistence → self-efficacy; Indirect effect 3 = Trait Mindfulness → Exercise motivation → exercise persistence → Self-efficacy. Significance levels: ***p < 0.001, **p < 0.01.

Path 1: Trait mindfulness → Exercise motivation → Self-efficacy (β = 0.224, 95% CI [0.176, 0.277]);

Path 2: Trait mindfulness → Exercise adherence → Self-efficacy (β = 0.100, 95% CI [0.067, 0.136]);

Path 3: Trait mindfulness → Exercise motivation → Exercise adherence → Self-efficacy (β = 0.072, 95% CI [0.050, 0.099]).

The respective contributions of the three indirect effects are 37.90, 16.69, and 12.18%, thereby validating hypotheses H2–H4.

## 4 Discussion

Based on the results and underlying assumptions, the main findings of this study can be summarized as follows:

Trait mindfulness exerts a direct influence on self-efficacy. The results confirmed that higher levels of trait mindfulness were associated with stronger self-efficacy among sports-disadvantaged students, consistent with previous research and theoretical expectations (Zhao et al., 2024). Exercise motivation and adherence serve as key mediators. The findings revealed that the positive effect of trait mindfulness on self-efficacy is not only direct but also indirect, functioning through increased exercise motivation and sustained exercise adherence (Sharp and Theiler, 2018). A multilayered psychological pathway is established. Together, these results extend current knowledge by demonstrating that mindfulness contributes to self-efficacy through a combination of motivational and behavioral mechanisms. This highlights new theoretical perspectives and practical implications for health promotion in physically inactive student populations.

### 4.1 Trait mindfulness and self-efficacy

The analysis first confirms that trait mindfulness positively predicts self-efficacy, consistent with earlier research (Li et al., 2022). Within the framework of Social Cognitive Theory, individuals’ beliefs in their capabilities are shaped by prior experiences and cognitive processing. Students with higher trait mindfulness are more likely to regulate emotions effectively and display greater cognitive flexibility (Fino et al., 2021; Goilean et al., 2023). These qualities help reduce anxiety and self-doubt during physical activity, thereby fostering mastery experiences that reinforce self-efficacy.

Beyond this, mindfulness has been described as a metacognitive process involving awareness and regulation of one’s own thinking (Thomson and Van Hedger, 2025). Empirical studies have shown that mindful agency predicts metacognitive ability, which in turn enhances self-leadership among student populations (Chen and Zhang, 2022). This suggests that metacognition may act as a pathway between mindfulness and self-efficacy: through improved awareness and cognitive control, individuals reinterpret challenges as opportunities for growth, further strengthening competence and confidence. Importantly, mindfulness also supports attentional control and reduces rumination, which is particularly relevant for sports-disadvantaged students who often begin with lower motivational baselines.

### 4.2 The mediating role of exercise motivation

Building on this direct relationship, the findings further indicate that exercise motivation serves as a partial mediator, adding nuance to the psychological processes involved. This result aligns with Self-Determination Theory, which emphasizes the importance of intrinsic motivation in sustaining health behavior. Mindfulness enhances individuals’ awareness during exercise and helps them align with their intrinsic goals (Jankauskiene and Baceviciene, 2024). For sports-disadvantaged students, who often lack external incentives or social support, this internalization of meaningful exercise goals is particularly critical (Cayleff, 1995).

Mindfulness promotes such internalization by fostering autonomy, a core element of SDT, and encouraging motivation that is self-endorsed rather than externally imposed (Ryan et al., 2021). In addition, it enhances perceived autonomy and competence—two essential needs within SDT—thereby facilitating the transition from controlled to autonomous forms of motivation (Aldbyani et al., 2024). Research in academic contexts has shown that mindfulness positively relates to self-efficacy, which in turn partly mediates its influence on performance outcomes (Aldbyani et al., 2024). Applied to exercise, this mechanism suggests that mindfulness enhances intrinsic motivation through strengthened self-efficacy, leading to deeper engagement and more sustained activity.

### 4.3 The mediating role of exercise adherence

Extending this pathway, exercise adherence emerged as another significant mediator. The Health Action Process Approach provides a useful framework for understanding this finding, as it highlights the transition from intention to long-term maintenance through planning and self-regulation. In this process, mindfulness strengthens volitional control, enabling individuals to manage fluctuations in motivation, cope with barriers, and maintain consistent routines. According to self-efficacy theory (Smith et al., 2023), repeated successful experiences reinforce personal agency (Xiang et al., 2024), and mindfulness contributes to this process by fostering non-judgmental acceptance and resilience in the face of setbacks (Eşkisu et al., 2020).

Mindfulness further enhances distress tolerance and pain endurance, both of which are essential for sustaining regular physical activity. Evidence shows that injured athletes receiving mindfulness training report higher pain tolerance, which supports rehabilitation and continued adherence (Mohammed et al., 2018). Likewise, mindfulness practices during exercise reduce perceived exertion, making sustained participation more manageable (Solk et al., 2023). Together, these findings demonstrate how mindfulness not only supports psychological resilience but also reduces subjective barriers, thereby reinforcing the adherence component of the pathway to self-efficacy.

### 4.4 Chain mediation mechanism

Taken together, the mediating roles of exercise motivation and adherence point to a chain mechanism through which trait mindfulness influences self-efficacy. Rather than acting in isolation, motivational quality (SDT) and behavioral persistence (HAPA) interact with cognitive belief formation (SCT) to form a comprehensive explanatory model of sustained health behavior. This integrated view demonstrates how mindfulness extends beyond momentary awareness, contributing to both the initiation and maintenance of physical activity, and ultimately strengthening self-efficacy.

Such an integrative framework represents a theoretical advancement by moving beyond single-pathway explanations. It also reflects recent calls for multi-theoretical health behavior models that offer greater explanatory power than approaches based on a single framework (Remskar et al., 2022). For example, interventions combining mindfulness and exercise have been shown to yield superior outcomes compared to either component alone (Tsang et al., 2008). By empirically validating a chain mediation model, this study enriches existing literature and provides practical implications for intervention design, especially for vulnerable student groups who face barriers to physical activity.

## 5 Limitations and future directions

This study provides meaningful insights into the interrelationships among trait mindfulness, exercise motivation, exercise adherence, and self-efficacy in sports-disadvantaged college students in China. Nonetheless, several limitations should be acknowledged, each of which offers opportunities for future research. The first limitation lies in the use of a cross-sectional design, which restricts the capacity to infer causality, as the observed associations may not capture the temporal sequence or directionality of effects. Future investigations should therefore consider adopting longitudinal or experimental designs to clarify causal pathways and determine the temporal ordering of trait mindfulness, exercise motivation, adherence, and self-efficacy (Neace et al., 2022).

Another limitation concerns the exclusive reliance on self-reported questionnaires, which raises the risk of response biases, including social desirability and self-protective tendencies, potentially compromising data accuracy. To mitigate such biases, future research should employ multi-method approaches that integrate objective behavioral indicators, observational ratings, or physiological measures, thereby enhancing measurement validity (Ortiz de Guinea et al., 2013). In addition, the sample in this study was confined to sports-disadvantaged college students in China, which restricts the generalizability of findings. Replication across more diverse cohorts—encompassing different age groups, athletic backgrounds, and cultural contexts—is necessary to strengthen external validity and ensure representativeness (Murphy et al., 2023).

Finally, cultural interpretations of trait mindfulness may vary across sociocultural settings. For example, while Western cultures often emphasize individual autonomy, Chinese culture tends to value collective orientation and social interconnectedness (Yi et al., 2021). Future research could therefore incorporate culturally relevant mediators—such as social support, communal values, or self-identity—to illuminate culturally contingent mechanisms linking mindfulness to self-efficacy. Addressing these limitations will contribute to a more nuanced theoretical framework and inform the design of culturally sensitive interventions in psychology, sports science, and education.

## 6 Conclusion

This study explored the chain mediation mechanism linking trait mindfulness to self-efficacy among sports-disadvantaged college students, with exercise motivation and adherence as key mediators. The findings show that mindfulness not only directly strengthens self-efficacy but also influences it indirectly through motivational and behavioral pathways. These results broaden the understanding of mindfulness and self-regulation by addressing an underrepresented group and suggest that interventions combining mindfulness practice with strategies to enhance motivation and adherence may be effective in promoting student well-being.

## Data Availability

The raw data supporting the conclusions of this article will be made available by the authors, without undue reservation.
