# Correction: Association between trait mindfulness and self-efficacy in sports-disadvantaged college students in China: the chain mediating role of exercise motivation and persistence

**DOI:** 10.3389/fpsyg.2025.1751383

**Published:** 2026-01-07

**Authors:** Kuo Xu, Lin Zhu, Yun Li, Yang Cao, Guodong Zhang

**Affiliations:** 1 Institute of Sport Science, College of Physical Education, Southwest University, Chongqing, China; 2 Biquan Primary School, Chongqing, China; 3 Clinical Epidemiology and Biostatistics, Faculty of Medicine and Health, School of Medical Sciences, Örebro, Sweden; 4 Unit of Integrative Epidemiology, Institute of Environmental Medicine, Karolinska Institutet, Stockholm, Sweden; 5 International College, Krirk University, Bangkok, Thailand

**Keywords:** exercise motivation, exercise persistence, trait mindfulness, self-efficacy, sports-disadvantaged college students, chain mediation effect

There was a mistake in Table 4 as published. The *p*-values were incorrectly formatted with an additional zero. The corrected Table 4 appears below.

**Table 4:** The mediating role of exercise motivation and persistence (*N* = 588).

**Model**	**Effect size**	**SE**	**95% Bootstrapping CI**	***p*-value**
Direct effect	0.195	0.036	0.125–0.266	<0.001
Total indirect	0.396	0.030	0.340–0.455	<0.001
Indirect effect 1	0.224	0.026	0.176–0.277	<0.001
Indirect effect 2	0.100	0.018	0.067–0.136	<0.001
Indirect effect 3	0.072	0.013	0.050–0.099	0.002
Total effect	0.591	0.034	0.525–0.658	<0.001

SE, standard error; Indirect effect 1 = trait mindfulness → Exercise motivation → self-efficacy; Indirect effect 2 = Trait mindfulness → Exercise persistence → self-efficacy; Indirect effect 3 = Trait Mindfulness → Exercise motivation → exercise persistence → Self-efficacy. Significance levels: ^***^p < 0.001, ^**^p < 0.01.

The original version of this article has been updated.

